# Accuracy and precision of few‐time‐points renal dosimetry for [^177^Lu]Lu‐PSMA‐617 therapy: Analysis with nonlinear mixed‐effects modeling

**DOI:** 10.1002/mp.70212

**Published:** 2025-12-25

**Authors:** Assyifa R. Hakim, Deni Hardiansyah, Elham Yousefzadeh‐Nowshahr, Ursula Nemer, Rien Ritawidya, Felix Kind, Ambros J. Beer, Philipp T. Meyer, Gerhard Glatting, Michael Mix

**Affiliations:** ^1^ Medical Physics and Biophysics Physics Department Faculty of Mathematics and Natural Sciences Universitas Indonesia Depok Indonesia; ^2^ Medical Radiation Physics Department of Nuclear Medicine Ulm University Ulm Germany; ^3^ Department of Nuclear Medicine Ulm University Ulm Germany; ^4^ Department of Nuclear Medicine Medical Center – University of Freiburg Faculty of Medicine University of Freiburg Freiburg Germany; ^5^ Research Center for Radioisotope Radiopharmaceutical, and Biodosimetry Technology National Research and Innovation Agency (BRIN) Tangerang Selatan Indonesia

**Keywords:** [^177^Lu]Lu‐PSMA‐617 therapy, FTP dosimetry, NLMEM

## Abstract

**Background:**

Determination of time‐integrated activity (TIA) with a reduced number of imaging sessions is essential for minimizing patient burden and clinical workload in the dosimetry of [^177^Lu]Lu‐PSMA‐617. One approach to achieve this is by performing single‐time‐point (STP) dosimetry. However, STP dosimetry may be associated with significant accuracy errors in certain patients, potentially impacting the reliability of dose calculations. Moreover, the assessment of precision is crucial to evaluating the stability and reliability of estimated doses.

**Purpose:**

This study aims to investigate the accuracy and precision of few‐time‐points (FTP) TIA calculation in kidneys for [^177^Lu]Lu‐PSMA‐617 using nonlinear mixed‐effects modeling (NLMEM).

**Methods:**

Biokinetic data of kidneys from 63 patients with metastatic castration‐resistant prostate cancer (mCRPC) treated with [^177^Lu]Lu‐PSMA‐617 in the first treatment cycle were used. The SPECT/CT measurement was done at time points (TP) 1) (1.8 ± 0.8) h, 2) (18.7 ± 0.9) h, 3) (42.6 ± 1.0) h, 4) (66.3 ± 0.9) h, and 5) (160.3 ± 24.2) h after injection. This study used the sum‐of‐exponentials function (SOEF) with six parameters, previously selected as the best fit function for the biokinetic data (PMID: 38423787). Reference TIAs (rTIAs) were derived from fitting the SOEF parameters to all‐time‐points (ATP) data within the NLMEM framework. Estimated TIAs (eTIAs) were calculated by fitting the FTP data, which consist of one‐, two‐, three‐, and four‐time points combinations of the biokinetic data. The accuracy of FTP‐NLMEM TIA calculations was quantified using the root‐mean‐square error (RMSE) and mean absolute percentage error (MAPE) of the relative deviation between eTIAs and rTIAs. Precision was assessed from the coefficient of variation (CV) of individual TIA estimates at each optimal time‐point combination.

**Results:**

For each optimal TP combination, the RMSEs and MAPEs were (11.0 ± 2.5)% and (7.0 ± 2.3)% for TP3, (6.3 ± 1.6)% and (4.8 ± 1.3)% for TP25, (3.9 ± 1.1)% and (2.3 ± 1.0)% for TP135, and (1.4 ± 0.8)% and (0.9 ± 0.8)% for TP1235. The %CV values of individual TIAs for each best TP combination were (17.1 ± 4.9)% for TP3, (8.5 ± 2.4)% for TP25, (6.3 ± 0.9)% for TP135, (5.1 ± 0.6)% for TP1235, and (4.4 ± 0.3)% for ATP.

**Conclusion:**

The accuracy and precision of various FTP schemes have been determined and can be used to decide on the number of measurements required. Our study showed that incorporating TP3 in FTP dosimetry could lead to a high accuracy and precision of calculated individual TIAs in [^177^Lu]Lu‐PSMA‐617therapy.

## 1 INTRODUCTION

In molecular radiotherapy (MRT), the organ absorbed dose is determined based on the time‐integrated activity (TIA) and the corresponding dose coefficient, accounting for energy deposition per unit activity.^1^ Individualized TIA calculation is beneficial as it allows for optimizing treatment while minimizing radiation exposure to organs at risk.^2^, ^3^ However, routine implementation in clinical practice is limited due to the need for multiple imaging sessions,^4^, ^5^ which increases patient burden and clinical workload. Therefore, developing accurate TIA estimation methods with fewer imaging sessions, e.g. with single‐time‐point (STP) imaging measurement, is of great interest.

The feasibility of STP dosimetry has been investigated for [^177^Lu]Lu‐PSMA‐617 therapy,^6^, ^7^, ^8^ peptide receptor radionuclide therapy (PRRT),^9^, ^10^ and radioiodine therapy.^11^, ^12^ One of the promising STP dosimetry methods for TIA estimation in [^177^Lu]Lu‐PSMA‐617 therapy is the population‐based model selection with nonlinear mixed‐effects modelling (PBMS NLMEM) approach.^2^ Although the NLMEM approach is not yet widely familiar within the nuclear medicine community, it constitutes standard practice in pharmaceutical sciences, where it is routinely applied for population analyses.^13^, ^14^, ^15^ NLMEM provides a major advantage for dosimetry by integrating population kinetics to constrain individual fits, thereby ensuring robust parameter estimation under sparse data conditions and accurately characterizing interpatient variability.^16^ A study involving 63 patients demonstrated that the PBMS NLMEM method could estimate kidney TIA from STP data with an average error of 10% at around 42 h after injection, outperforming frequently used monoexponential STP methods by Hänscheid et al.^9^ and Madsen et al.^10^ However, the PBMS NLMEM method produced considerable errors (up to 39%) in certain patients, mainly those exhibiting low biological clearance in the population. Thus, balancing accuracy and reduced imaging sessions remains a critical challenge.

In clinical practice, the challenge of balancing accuracy and reduced imaging sessions in STP dosimetry is further compounded by the occasional inability to perform imaging at a specific time point due to patient‐related factors, necessitating alternative strategies for TIA estimation. Therefore, assessing how the exclusion of time points impacts TIA accuracy and determining the feasibility of alternative sampling schedules is crucial. This underscores the need for adaptable few‐time‐point (FTP) dosimetry approaches that can accommodate clinical constraints while preserving the accuracy of TIA calculations.

In addition to TIA accuracy, the precision of individual TIA estimates is another recommended parameter to be calculated in MRT dosimetry. The precision of individual TIA calculations using FTP dosimetry with PBMS NLMEM for [^177^Lu]Lu‐PSMA‐617 has not been previously reported in the literature. This information is highly relevant, which is why the European Association of Nuclear Medicine (EANM) guidelines recommend the calculation of the standard deviation (SD) of individual TIA for precision analysis in MRT.^17^ Protocols incorporating reduced imaging schedules in MRT often suggested that the first cycle be characterized with all time‐point (ATP) biokinetic data, whereas subsequent cycles use reduced time‐points, e.g., STP dosimetry.^18^ However, without knowledge of the TIA's SD, it becomes challenging to determine whether TIA values remain stable, increase, or decrease across treatment cycles. Including the SD of individual TIAs as a measure of precision could be a valuable quality‐control tool.

Recently, FTP dosimetry with PBMS NLMEM has been applied to assess TIA accuracy in PRRT using [^111^In]In‐DOTA‐TATE biokinetic data acquired by planar scintigraphy in eight patients as surrogate for [⁹⁰Y]Y‐DOTA‐TATE.^19^ However, to the best of our knowledge, no study has evaluated the accuracy and precision of FTP dosimetry with PBMS NLMEM for [^177^Lu]Lu‐PSMA‐617 using 3D SPECT/CT‐derived biokinetic data. Therefore, this study aims to evaluate the accuracy and precision of FTP renal TIA calculation using the PBMS NLMEM method for [^177^Lu]Lu‐PSMA‐617 therapy of metastatic castration‐resistant prostate cancer (mCRPC). The analysis is conducted on a large cohort of 63 patients, using 3D SPECT/CT biokinetic data with a broad range of measured time points, i.e., 0.69 to 235.66 h after injection.

## 2 MATERIALS AND METHODS

### 2.1 Biokinetic data

This study used biokinetic data of [^177^Lu]Lu‐PSMA‐617 in the kidneys of 63 patients with mCRPC in the first treatment cycle.^20^ The patients were administered a median of 6 GBq/cycle of [^177^Lu]Lu‐PSMA‐617 activity intravenously. Following clinical standard operating procedure, SPECT/CT measurements at time points TP1 = (1.8 ± 0.8) h, TP2 = (18.7 ± 0.9) h, TP3 = (42.6 ± 1.0) h, TP4 = (66.3 ± 0.9) h, and TP5 = (160.3 ± 24.2) h of SPECT/CT after the injection were performed. Written informed consent was obtained from all subjects, and the institutional review board (vote no. 326/18) approved the retrospective study.

### 2.2 Calibration and phantom study

A dual‐head SPECT/CT system (BrightView XCT; Philips Healthcare) equipped with 3/8‐inch NaI(Tl) detectors and a medium‐energy general‐purpose collimator was used for data acquisition. Imaging was performed with 40 projections of 20 s per head in a body‐contour orbit, using a 128 × 128 matrix. An energy window of ±10% centered on the 208‐keV photopeak was used. Attenuation correction was based on low‐dose cone‐beam CT scans acquired at 120 kVp and 30 mAs. The scatter was corrected using the vendor's effective scatter source estimation method during image reconstruction.^21^

SPECT images were reconstructed using an ordered‐subsets expectation maximization (OSEM) algorithm with 4 iterations and 16 subsets. A Butterworth postreconstruction filter (cutoff frequency 0.4, order 1.4) was applied. Measurements were performed using the National Electrical Manufacturers Association (NEMA) NU‐2 2007 body phantom, following the same acquisition and reconstruction protocol as described above. The phantom body was filled with water, and two kidney‐shaped inserts (500 mL each) were positioned, containing 100 and 200 MBq of ^177^Lu, respectively. Calibration measurements were repeated four times at two‐day intervals, and the calibration factor for the imaging protocol, calculated as the mean of both kidney inserts across all measurements,^20^ was (9.9 ± 0.4) cps/MBq. Resolution recovery was not implemented to avoid edge artifacts.^20^

### 2.3 Kidney segmentation and quantification

The software Rover ABX (ABX Advanced Biochemical Compounds GmbH) was used for kidney segmentation and analysis.^22^ CT scans were used to delineate the kidneys, and within each TP, activity was measured in both the left and right kidneys. All kidney volumes of interest were expanded by 2 SPECT voxels in all directions to compensate for spill‐out effects arising from the limited spatial resolution of the SPECT system. The two‐voxel margin was determined from phantom measurements to achieve full activity recovery of the kidney inserts after applying the calibration factor. Finally, this study used the fraction of injected activity in both kidneys.

### 2.4 Uncertainty considerations

In addition to attenuation and scatter correction, several further aspects influence the quantitative accuracy of SPECT/CT‐based activity measurements. The uncertainties associated with attenuation and scatter correction in the employed system have been reported to be below 10%.^23^ The stability of the calibration factor was maintained within 5% as verified by routine weekly quality control of the detection efficiency. For kidney activity measurements, partial‐volume effects were compensated by applying a phantom‐derived enlargement factor, yielding a residual inaccuracy of approximately 5%. The predominant source of quantitative uncertainty originated from statistical noise in the reconstructed SPECT images, which was particularly relevant at later time points, with standard deviations of 15%–20% for the mean activity values within the defined volumes of interest.

### 2.5 Study workflow

NONMEM software (Version 7.5, ICON Development Solutions, USA) was used to perform the NLMEM fittings and the simulations. All fitting was performed with the NLMEM framework using the Laplacian First‐Order Conditional Estimation Method (FOCE), where the optimal starting value is obtained via the Markov Chain method. The analyses were performed using Python 3.11 programming language with Spyder IDE (Version 1.4.3, https://www.spyder‐ide.org/) within the Anaconda (Version 2023.07‐2, Anaconda, Inc) environment. The Laplacian FOCE method was used in this study to enable direct comparison with our previous SAEM‐based analysis.

The best sum‐of‐exponentials function (SOEF) identified for the kidney biokinetic data of [^177^Lu]Lu‐PSMA‐617 for this patient population^6^ was used:

(1) $$\begin{array}{cc} f\;\left(t\right) & =A_{1}\;e^{-\left(\lambda_{1}+\lambda_{phys}\right)t}+A_{2}e^{-\left(\lambda_{2}+\lambda_{phys}\right)t}-A_{3}e^{-\left(\lambda_{3}+\lambda_{phys}\right)t}\\ & \;-\left(A_{1}+A_{2}-A_{3}\right)e^{-\left(\lambda_{bc}+\lambda_{phys}\right)t} \end{array}$$

where the $A_{i}$ (i=1,2,and3) are the prefactors, $\lambda_{i}$ (i=1,2,and3) are the biological uptake or clearance rates of the radiopharmaceutical, $\lambda_{phys}$ is the physical decay constant of ^177^Lu which is $\frac{\ln(2)}{T_{\frac{1}{2}}}$ where $T_{\frac{1}{2}}$ is the half‐life of ^177^Lu (6.6443 days),^24^ and $\lambda_{bc}$ is the rate due to blood circulation uptake with half‐life of 1 min $\left(\frac{\ln(2)}{1\;min}\right)$.^2^ The structural parameters ($A_{i}$ and $\lambda_{i}$) are constrained to values greater than zero.

In the NLMEM, parameter estimation was performed at the population level. Each SOEF parameter was assigned a typical value parameter representing the population mean (the fixed effect parameter), while interindividual variability was modeled as random effects. In addition, a residual error term was estimated to account for intraindividual variability and measurement error. For the six parameters of the SOEF model, six fixed effects, six random effects, and one residual error term were estimated, resulting in a total of 13 parameters. Because the fitting was conducted population‐wise, data from all subjects (e.g., 63 patients) were analyzed jointly, ensuring that the total number of observations exceeded the number of estimated parameters and thus satisfied the degrees of freedom requirement. Further explanation about the NLMEM fitting is available in Supplemental Section A.

The TIA can be calculated analytically by taking the integral of Equation (1) along the time domain (from zero to infinity)

(2) $$\begin{array}{cc} TIA & =\frac{A_{1}}{\lambda_{1}+\lambda_{phys}}+\frac{A_{2}}{\lambda_{2}+\lambda_{phys}}-\frac{A_{3}}{\lambda_{3}+\lambda_{phys}}\\ & \;-\frac{\left(A_{1}+A_{2}-A_{3}\right)}{\lambda_{bc}+\lambda_{phys}} \end{array}$$

Each parameter's SD was derived from the square root of the corresponding diagonal element of the covariance matrix obtained from the model fitting. The covariance of the parameters was then propagated to estimate the SD of the TIA. The entire error propagation procedure was performed within the NONMEM software environment.

**FIGURE 1:**
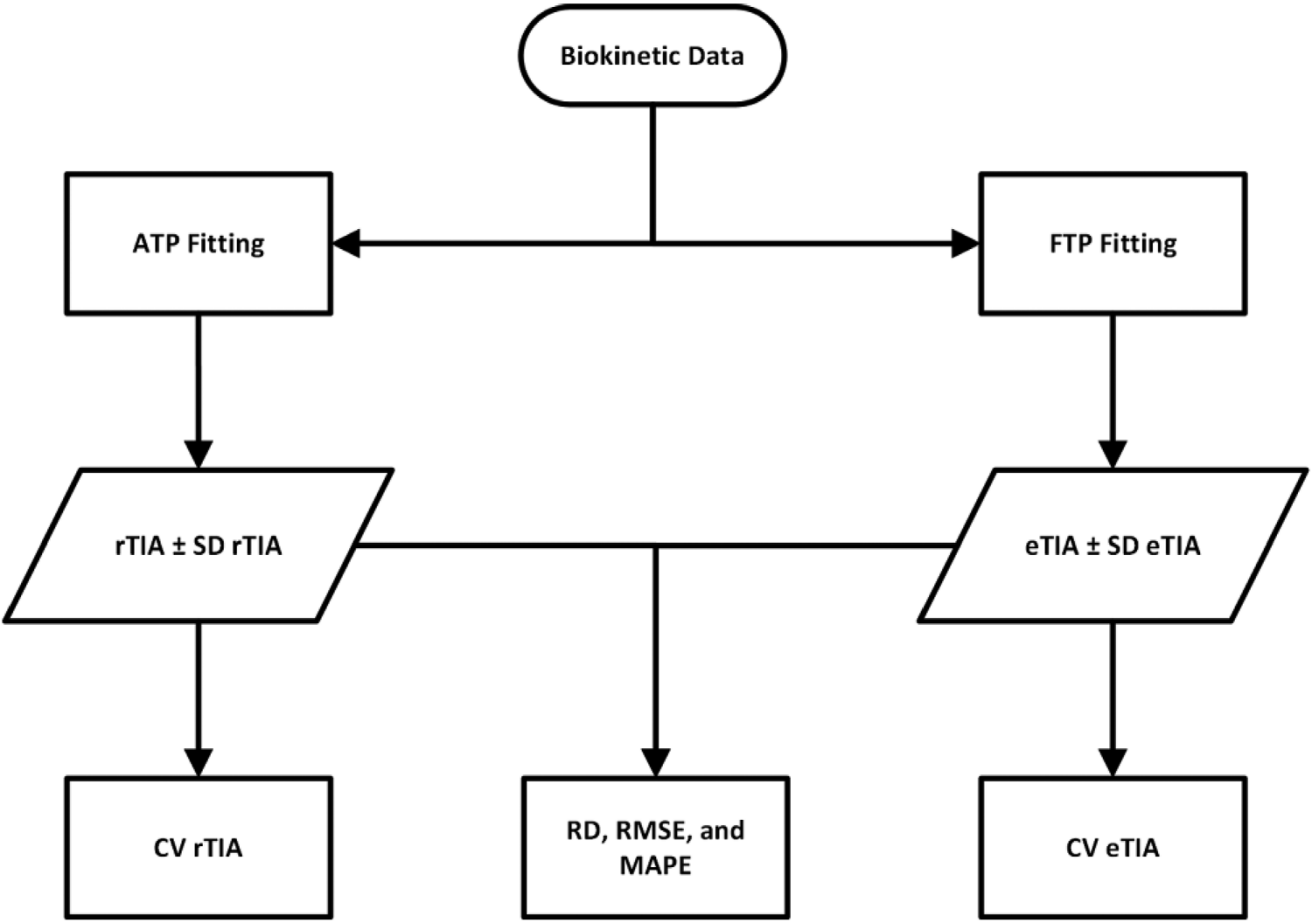
Workflow of the study. ATP with NLMEM fitting was performed to obtain the rTIA along with its SD. FTP with NLMEM fitting was then performed to obtain the eTIA and its SD. The TIA and its SDs were used to calculate the CV for each TIA, which was further used to analyze the precision of the TIA calculations. The RD and RMSE between rTIA and eTIA were then calculated to analyze the accuracy of the TIA calculations. ATP, all‐time‐points; CV = Coefficient of Variation; eTIA = estimated TIA; FTP, few‐time‐points; MAPE, mean absolute percentage error; RD, relative deviation, RMSE, root‐mean‐square error, rTIA, reference TIA; SD, standard deviation.

The workflow of this study is illustrated in Figure 1. The reference TIA (rTIA) and its standard deviation (SD rTIA) were obtained by fitting the SOEF parameters from Equation (1) to ATP data in each patient (Section 2.1). The estimated TIA (eTIA) and its standard deviation (SD eTIA) were derived by fitting the biokinetic data using the few‐time‐point (FTP) method, considering combinations of STP, two‐time‐points (2TP), three‐time‐points (3TP), and four‐time‐points (4TP) datasets. Additionally, to mimic a fixed‐activity strategy, we evaluated a no‐time‐point (NTP) dosimetry approach in which no individual imaging or time‐activity data are acquired. Instead, the eTIA is estimated for each patient using the population‐derived SOEF parameters of all patients, applying either the mean (NTPme) or the median (NTPmd) of the individual parameters. Thus, in this surrogate approach, all patients are assigned the same representative parameter values and eTIA value.

The accuracy of eTIA estimates was assessed by computing the relative deviations (RDs), the root‐mean‐square errors (RMSEs), and the mean absolute percentage errors (MAPEs), using rTIA as the reference:

(3) $$\;RD_{k,m}=\frac{eTIA_{k,m}-rTIA_{m}}{rTIA_{m}}\;$$

(4) $$\;RMSE_{k}=\sqrt{{\left(SDRD_{k}\right)}^{2}+{\left(MeanRD_{k}\right)}^{2}\;}\;$$

(5) $$\;\mathrm{MAP}E_{k}=\frac{100}{N}\;\sum_{m=1}^{N}\left|\frac{\mathrm{eTI}A_{k,m}-\mathrm{rTI}A_{m}}{\mathrm{rTI}A_{m}}\right|$$

where $RD_{k,m}$ is the RD of dosimetry method k (e.g. NTP, STP, 2TP, 3TP, or 4TP) of patient m, $eTIA_{k,m}$ is the eTIA with the method k of patient m, $rTIA_{m}$ is the rTIA of patient m, $RMSE_{k}$ is the RMSE of method k over all patients, $SDRD_{k}$ is the standard deviation of RD of method k over all patients, $MeanRD_{k}$ is the average of the relative deviation with method k over all patients, and $MAPE_{k}$ is the mean absolute percentage error of method k over all N patients. The best TP combination was identified as the one yielding the highest accuracy, corresponding to the lowest values of both RMSE and MAPE. Both $rTIA_{m}$ and $eTIA_{k,m}$ have associated SD values. Accordingly, the SD of each individual $RD_{k,m}$ was obtained by error propagation. Based on these values, the SDs of the RMSE and MAPE were subsequently derived by propagating the corresponding uncertainties. Explanation about SD calculation of RMSE and MAPE is available in Supplemental Section B.

Both the RMSE and the MAPE were calculated with the leave‐one‐out (Jackknife) method for all combinations of TPs. The total number of patients with RD > 10% (RD10) and RD > 20% (RD20) were also analyzed for all NTP and FTP combinations. RMSE, MAPE, RD10, and RD20 were used as accuracy parameters of the TIAs for NTP and FTP dosimetry. Simple linear regression between eTIA and rTIA was performed using GraphPad Prism version 10.6.0 for Windows (GraphPad Software, Boston, Massachusetts USA, www.graphpad.com). A *p*‐value < 0.05 was considered statistically significant for testing whether the regression slope differed from zero.

All the acquired individual TIAs (rTIAs and eTIAs) and their corresponding standard deviation SD were used to calculate the individual TIA precision as the Coefficient of Variation (CV) with the following equation:

(6) $$CV=\frac{SD_{TIA}}{Mean_{TIA}}\;$$

where the CVs are calculated for each patient TIA in each method. The CVs were used as precision parameters of the individual TIAs for ATP and FTP dosimetry.

## 3 RESULTS

**FIGURE 2:**
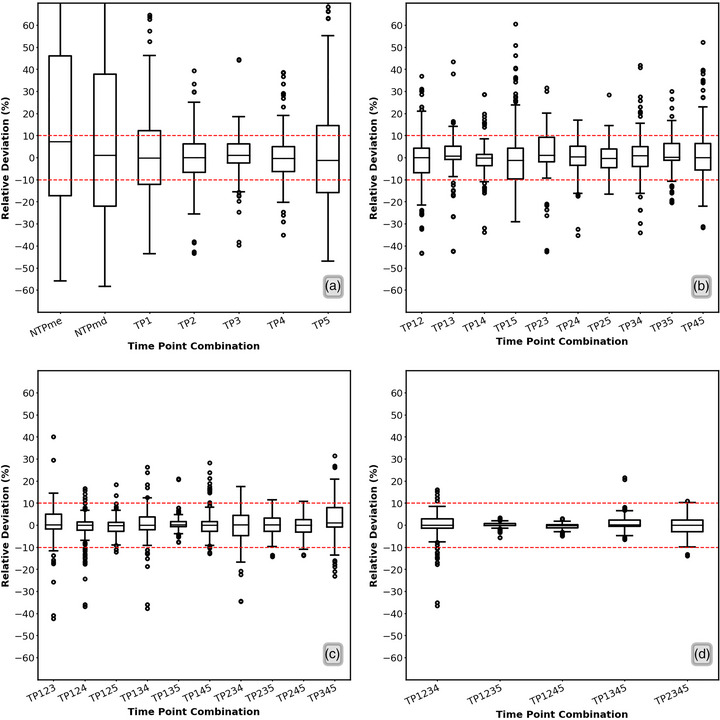
Boxplot of RD between the eTIA from NTP and each TP combination of FTP NLMEM fitting and rTIA from ATP NLMEM fitting. (a) The boxplot of the RD from NTPme, NTPmd, and STP dosimetry, (b) the boxplot of RD from 2TP dosimetry, (c) the boxplot of RD from 3TP dosimetry, and (d) the boxplot of RD from 4TP dosimetry.

Table 1 shows the RD distribution for the optimal FTP combination of STP, 2TP, 3TP, 4TP, and NTP, along with their RMSEs and MAPEs and their respective uncertainties. Figure 2 shows the boxplots of the RDs of all the FTP combinations. The NTP method showed a much higher RMSE and MAPE for eTIA values than that of FTP dosimetry (Table 1 and Figure 2). For STP, the best TP combination with the lowest RMSE among all STP combinations was TP3, which has an RMSE value of (11.0 ± 2.5)%. For 2TP, the best TP combination was TP25, which has an RMSE value of (6.3 ± 1.6)%, the lowest among all 2TP combinations.

**TABLE 1:** Accuracy (RMSEs, MAPEs, RD10, and RD20) of eTIAs from NTP and best TP combination of FTP with NLMEM TIA calculations. Standard deviations (SDs) of RMSE and MAPE were derived by error propagation from individual RD values. RD10 and RD20 represent the counts of patients with RD values exceeding 10% and 20%, respectively, with the values in parentheses indicating the percentage of patients above each threshold.

Optimal combination of time points	RD median [Min, Max]	RMSE (SD)	MAPE (SD)	RD10 (%)	RD20 (%)
NTPme	7.2 [−55.7, 303.8]	65.3 (9.3)	42.2 (4.8)	52 (83)	34 (54)
NTPmd	1.0 [−58.3, 280.6]	59.8 (2.5)	39.4 (1.2)	55 (87)	39 (62)
TP3	0.4 [−39.0, 44.2]	11.0 (2.5)	7.0 (2.3)	15 (24)	3 (5)
TP25	−0.3 [−14.9, 17.5]	6.3 (1.6)	4.8 (1.3)	5 (8)	0 (0)
TP135	0.1 [−7.6, 20.7]	3.9 (1.1)	2.3 (1.0)	1 (2)	1 (2)
TP1235	0.0 [−5.7, 2.7]	1.4 (0.8)	0.9 (0.8)	0 (0)	0 (0)

**FIGURE 3:**
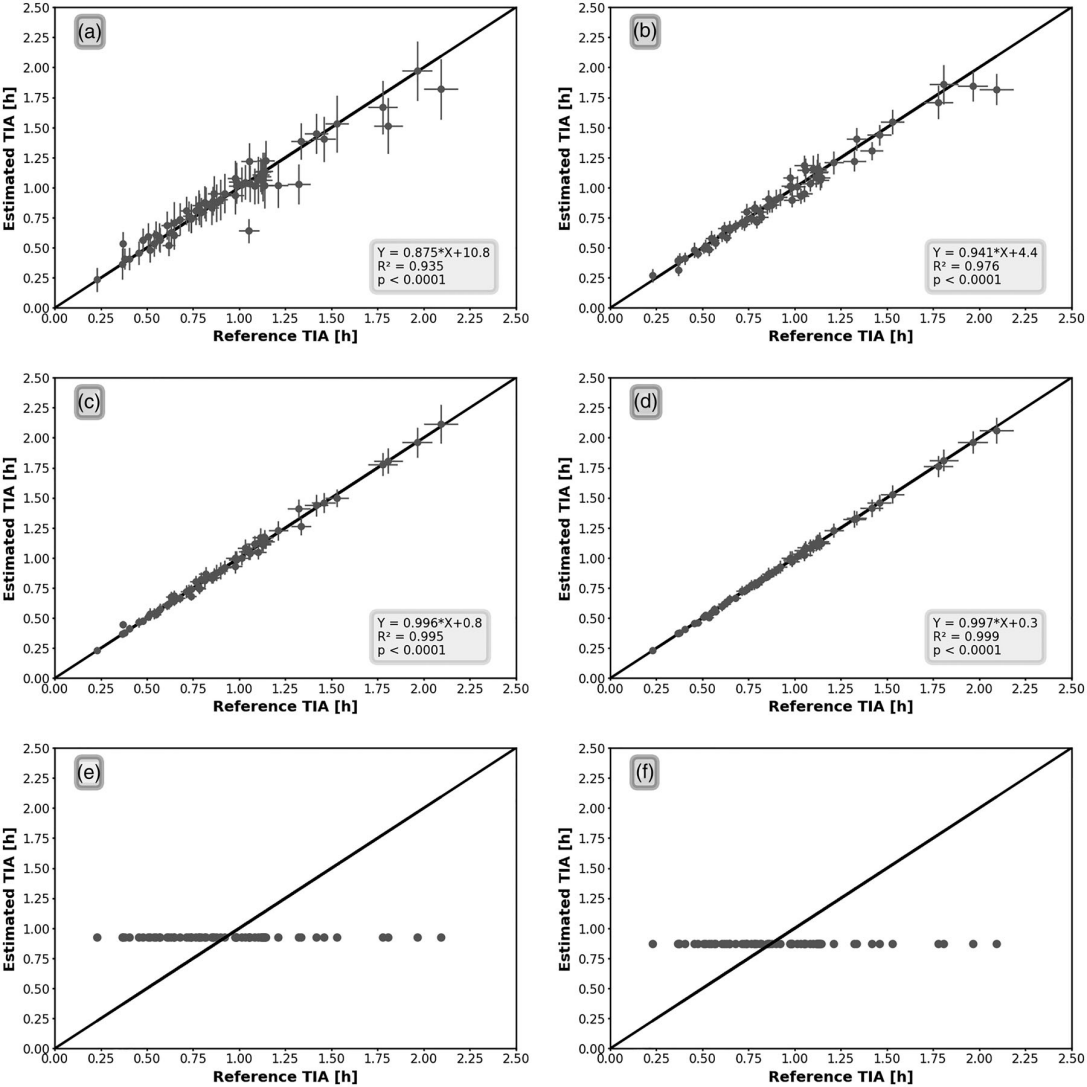
Relationships (accuracy and precision) between the reference and estimated TIA for the best combination of (a) STP (TP3), (b) 2TP (TP25), (c) 3TP (TP135), (d) 4TP (TP1235), (e) NTPme, and (f) NTPmd dosimetry.

**FIGURE 4:**
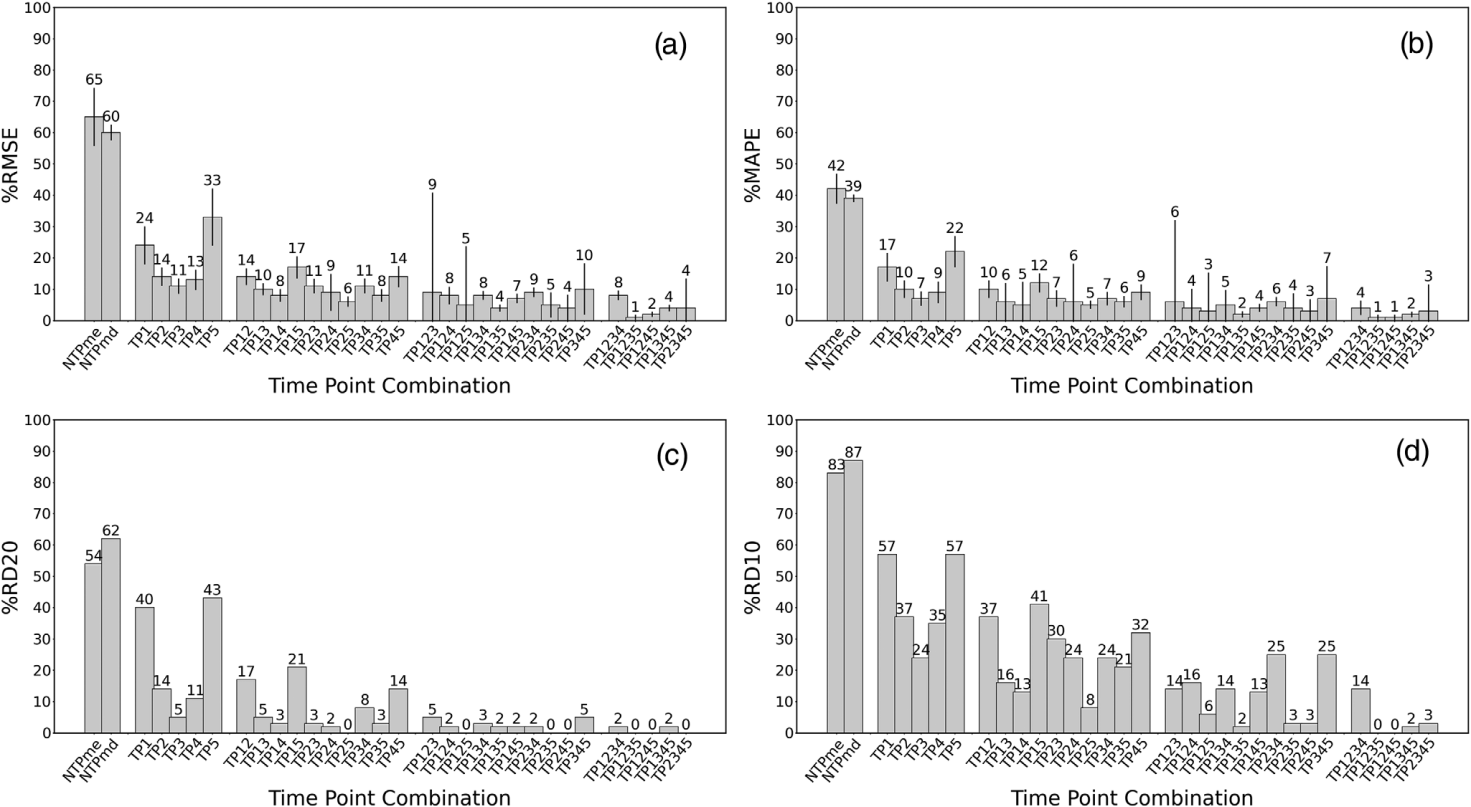
Bar chart showing the accuracy of NTP and FTP dosimetry as the percentage of patients with (a) %RMSE, (b) %MAPE, (c) RD20, and (d) RD10 for each schedule. The schedules are ordered according to the number of TPs used.

The 2TP combination of TP35 also resulted in an acceptable RMSE with a value of (8.4 ± 2.0)%. This result suggested that including TP3, which was identified as the TP that could lead to the most accurate STP dosimetry, may increase the accuracy of TIA calculation in 2TP dosimetry. The best 3TP and 4TP combinations were TP135 and TP1235, which have RMSE values of (3.9 ± 1.1)% and (1.4 ± 0.8)%, respectively. These results also confirmed the importance of including TP3 to 3TP and 4TP dosimetry. Figure 3 shows the accuracy and precision of the eTIA based on the rTIA values for the best combinations of STP, 2TP, 3TP, and 4TP dosimetry. The *p*‐value (Figure 3) confirms the presence of a statistically significant correlation between eTIA and rTIA across all methods, supporting the validity of the FTP dosimetry approach. The accuracy (Figure 4) and precision (Tables 1 and 2) of eTIA are improving with the total number of data points used for the dosimetry.

**TABLE 2:** Precision (distribution of the coefficients of variation CV) of estimated time‐integrated activities (TIA) from all‐time‐points non‐linear mixed‐effects modelling (NLMEM) TIA calculations, and the best time point of few‐time‐point NLMEM TIA calculations.

	CV of the individual TIA (%)	
Optimal combination of time points	Mean (SD)	Median [Min, Max]
ATP	4.4 (0.3)	4.3 [4.1, 5.6]
TP1235	5.1 (0.6)	5.1 [4.0, 6.4]
TP135	6.3 (0.9)	6.2 [4.9, 9.8]
TP25	8.5 (2.4)	7.8 [5.7, 20.7]
TP3	17.1 (4.9)	16.1 [10.9, 42.4]

Table 2 shows the CV value of individual TIAs for ATP and all best FTP combinations. In general, FTP dosimetry may result in acceptable precision. Still, it should be performed with caution, especially for STP, as the CV value of individual TIA could be around three times that of the ATP. The 2TP combination of TP35 shown in Table 2 resulted in individual TIAs with CV values of (12.4 ± 3.0)%.

## 4 DISCUSSION

### 4.1 Key findings on accuracy and precision

In this study, we analyzed the accuracy and precision of kidney TIA calculation with FTP and the PBMS NLMEM method from 63 patients treated with [^177^Lu]Lu‐PSMA‐617. RMSE and MAPE are used to represent accuracy for each method, while the CV of the individual TIA represents the precision for the TP combination. The accuracy of eTIAs is regarded as acceptable if the value is below 10%, whereas the precision of eTIAs is considered precise if the value is below 25% and acceptable if the value is below 50%.^25^

Our results show that the best TP for the STP dosimetry method is TP3 (42.6 ± 1.0) h after injection. This result is in agreement with our previous study in which the biokinetic data are the same.^6^ There is a marginal difference between the TIA's accuracy results of the STP dosimetry in this to that in our previous study, e.g. RMSE = 11% in this study vs RMSE = 10% in previous study^6^ at TP3, that could be apportioned to the different software used for the analysis (NONMEM in this study vs MATLAB in previous study^6^), fitting algorithm (FOCE in this study vs SAEM in previous study^6^), and the method used for determining the optimal starting value of NLMEM fitting (Markov Chain in this study vs random number drawing in previous study^6^). Both SAEM and FOCE have been shown to yield similar results for the study of nosocomial infections and piperacillin pharmacokinetics.^26^ Our findings similarly indicate marginal differences between the two approaches, highlighting their interchangeability and practical value for biokinetic modeling in renal dosimetry of [^177^Lu]Lu‐PSMA‐617 therapy.

The results indicate that the STP method yields the lowest accuracy among FTP dosimetry approaches, whereas 4TP dosimetry achieves the highest accuracy. This finding suggests that incorporating additional data into FTP TIA calculations enhances accuracy, consistent with a previous FTP dosimetry study in radionuclide therapy^19^ and in line with studies of multiple but fewer time points.^27^ Nevertheless, the comparison with the eTIAs from the NTP case, which uses a mean NTPme and median value NTPmd for all patients, demonstrates that even the STP approach dramatically enhances the accuracy of the personalized TIA (Table 1).

Although TP3 for the STP TIA calculation provides acceptable accuracy, it is important to note that the RD can exceed 30% in some patients. Including additional data in the calculation of eTIAs could help lower RDs, particularly in outlier patients identified in our previous study.^6^ Specifically, for the optimal time point combination in FTP dosimetry, the RDs in the same outlier patient were observed to be as high as 39%, 12%, 7%, and 1% for STP, 2TP, 3TP, and 4TP dosimetry, respectively.

The precision of eTIAs follows a trend similar to that of accuracy. As shown in Table 2, increasing the total number of data points reduces the CV in individual TIA calculations, thereby improving TIA's precision. All CVs obtained from ATP, 4TP, 3TP, and 2TP dosimetry methods at the optimal time point combinations are below 25%, falling within the precise range.^25^ STP dosimetry leads to precisions with mean ± SD of CV of (17.1 ± 4.9)% at TP3. However, in some patients in STP dosimetry, the CV of eTIAs can reach 42.4%, though it remains within the acceptable range of precision criteria.^25^

Our results demonstrate that incorporating additional imaging data into few‐time‐point (FTP) TIA calculations, e.g., from 2TP to 4TP, substantially enhances accuracy, yielding approximately a twofold reduction in estimated MAPE at the best TP combination (Table 1). Similarly, increasing the number of time points from 2TP to 4TP reduced the mean CV in individual TIA estimates by approximately 50% (Table 2).

### 4.2 Clinical and practical implications

Overall, increasing the number of imaging time points in FTP dosimetry improves both the accuracy and precision of predicted TIAs. However, the trade‐off between the number of required imaging sessions and the achievable accuracy and precision must be carefully considered in clinical decision‐making. Supplemental Table S1 and Figure 4 provide a practical framework to guide this process, enabling clinicians to select optimal measurement schedules based on predefined accuracy and precision requirements when a patient cannot undergo the complete recommended set of assessments. For example, if only three measurements are feasible, the optimal time points can be chosen to maximize accuracy and precision while maintaining clinical relevance.

The results show that TP3, identified as the best TP for STP dosimetry, also contributes substantially to TIA calculations across all FTP combinations. For example, TP135 and TP1235 are identified as the best time point combinations for 3TP and 4TP TIA calculations, respectively. Although the best TP combination for 2TP TIA calculations is TP25 (Table S1), TP35 also provides acceptable accuracy, with RMSE (8.4 ± 2.0)% and MAPE (5.8 ± 1.8)%. Table S1 also shows the precision of TP35 within the defined criteria, with the CV of individual eTIAs of (12.4 ± 3.0)%. However, some patients could have 24.4%, which is still within an acceptable range.

This approach is particularly useful when imaging at TP2 is not feasible due to safety or logistical constraints. In such cases, clinicians may select TP3 as an alternative while still maintaining an acceptable balance between accuracy and precision. By strategically selecting measurement time points, clinicians can obtain meaningful and reliable insights, thereby enhancing the clinical applicability of FTP dosimetry.

### 4.3 Comparison with prior work and methods

In terms of STP, several simple methods are available, such as those from Hänscheid et al.^9^ and Madsen et al.^10^ Our previous study with similar datasets compared the accuracy of these two methods and NLMEM at 42.6 ± 1.0 h post‐injection (TP3 in this study). The RMSE of STP with NLMEM was 10%, while the methods of Hänscheid et al. and Madsen et al. yielded RMSE values of 34% and 21%, respectively.^6^ Another study showed similar results for [^177^Lu]Lu‐DOTA‐TATE.^28^ Moreover, these studies showed that NLMEM reduced bias and variance, and it also improved the robustness of STP dosimetry. NLMEM also demonstrated potential for handling sparse or unbalanced population data.^26^ This comparison highlights that the PBMS NLMEM approach consistently outperforms simplified STP methods and maintains high accuracy even under conditions of reduced data availability, reinforcing its suitability for clinical and quantitative medical physics applications.

### 4.4 NLMEM‐specific considerations

The NLMEM approach utilizes prior knowledge from a previous population to refine eTIA calculations for individual patients, thereby enhancing the accuracy of individual dosimetry. Unlike the NTP approach, which assumes identical kinetics across all patients and thus neglects individual variations in uptake and clearance rates, the FTP approach incorporates one to five patient‐specific measurements to estimate biokinetic parameters individually. This personalization markedly enhances TIA estimation accuracy (yielding lower RMSE and MAPE), highlighting the meaningful impact of even limited patient‐specific data on reliable dosimetry.

A leave‐one‐out methodology was employed in this study as part of the proposed FTP dosimetry approach. Although commonly used for model validation, this technique carries the risk of overfitting, particularly in models with strong nonlinearity or when the number of parameters is large relative to the dataset size.^29^, ^30^ However, the model derived from our PBMS NLMEM analysis is relatively simple, comprising only thirteen parameters: six fixed effects, six inter‐individual variability components, and one residual variability component. NLMEM performed population‐wise fitting, so it was performed for all patients simultaneously. Therefore, in this case, for all 63 patients with 5 time points each, the total number of data points will be 63×5=315. The modest parameter count relative to the 315 analyzed data points minimizes the risk of overfitting and supports the robustness of the results. Statistically, this satisfies the requirement K+1≤N for all FTP dosimetry scenarios. Table S1 shows the number of data points per estimated parameter for all FTP dosimetry scenarios.

Moreover, unlike machine learning approaches, our method incorporates prior knowledge of the functional form of the fitting function.^25^, ^31^ This built‐in structure, combined with the model's low complexity, helps mitigate the potential for strong non‐linearity and further reduces the likelihood of overfitting. Although alternative validation techniques such as k‐fold cross‐validation might yield slightly different outcomes or offer deeper insights into model robustness, they were not included in this study due to scope limitations. Future investigations could benefit from integrating these methods to more thoroughly assess overfitting risks and strengthen confidence in model reliability.

Although the PBMS NLMEM approach for STP dosimetry requires expertise in nonlinear modeling and model selection, it represents an established and regulatory‐endorsed methodology—recognized by both the FDA and EMA as the standard framework for population analyses in drug development and pharmacokinetic modelling.^13^, ^14^, ^15^, ^16^ This endorsement underscores the robustness of the approach and highlights that methodological complexity should not be viewed as a limitation when it reflects best scientific practice. The NLMEM framework effectively accounts for interpatient variability by simultaneously estimating individual parameters and their population distributions, thereby enhancing model accuracy and clinical relevance. Although specialized expertise and computational resources are often required during model development, the application of PBMS NLMEM becomes relatively straightforward once an appropriate model has been established. Moreover, the growing availability of user‐friendly software tools and the development of interfaces tailored for clinical dosimetry are expected to reduce technical barriers and facilitate broader clinical implementation. Although the present study focuses on a single radionuclide, the proposed framework is generalizable and should be further validated across different therapeutic radionuclides to confirm its robustness and clinical applicability.

### 4.5 Future applications

In clinical practice, implementing NLMEM with an extensive dataset of historical [^177^Lu]Lu‐PSMA‐617 biokinetic data employing multiple time points is essential for optimizing TIA (and, subsequently, absorbed dose) estimation. Although collecting biokinetic data to establish a comprehensive population database is a time‐intensive process, facilitating data exchange between healthcare centers—supported by the insights provided in this study—can significantly accelerate this effort, ultimately improving personalized dosimetry and clinical outcomes.

Adopting FTP dosimetry (e.g., 3TP or 4TP) protocols would further streamline clinical dosimetry workflows by reducing the number of imaging sessions, scanner occupancy, and patient burden compared with 5TP protocols, thereby improving feasibility in routine clinical practice. However, this study focuses on evaluating the accuracy and precision of TIA estimation under reduced sampling conditions, without analyzing the relationship between TIA prediction accuracy and patient outcomes, which will be the subject of future investigations.

Dosimetry of [^177^Lu]Lu‐PSMA‐617 using the FTP and PBMS NLMEM approaches might also be helpful for tumor or other organ dosimetry. Model selection procedures can be used to identify the SOEF model that best describes radiopharmaceutical kinetics in tumors or other organs. Once selected, the TIA can be calculated by analytically integrating the SOEF model, with uncertainty estimated by propagating the parameter standard deviations. The NLMEM framework is particularly valuable for quantifying interpatient variability and assessing differences among individual tumors or other organs.

Nonetheless, further studies are required to evaluate the accuracy and precision of FTP dosimetry for tumors or other organs. Given the differing kinetic profiles of radiopharmaceuticals in kidneys, tumors, or other organs, the optimal SOEF models, estimated residual variability, and parameter values may differ. Although the uncertainty‐propagation methodology would remain similar, the magnitude of uncertainty could be higher in tumors than in kidneys.

## 5 CONCLUSIONS

This study is the first that assessed both the accuracy and precision of various FTP combinations for TIA calculations of [^177^Lu]Lu‐PSMA‐617 in the kidneys using the PBMS NLMEM method. Our findings indicate that fewer data points may compromise the accuracy and precision of TIAs. However, the FTP with NLMEM method demonstrated acceptable performance in accuracy and precision, particularly when incorporating biokinetic data at TP3 (42.6 ± 1.0 h after injection). Implementing this FTP with the NLMEM approach in clinical practice could enhance the effectiveness of [^177^Lu]Lu‐PSMA‐617 therapy by contributing to optimized, patient‐specific dosimetry, thereby supporting enhanced treatment outcomes.

## CONSENT FOR PUBLICATION

All authors read the manuscript and consented to its publication.

## CONFLICT OF INTEREST STATEMENT

Deni Hardiansyah and Gerhard Glatting serve as independent consultants for ITM Oncologics GmbH. The authors declare that they have no competing interests.

## Supporting information



Supporting Information

## Data Availability

The data that support the findings of this study are available from Michael Mix upon reasonable request.
