# Supplementary material for: Accuracy and precision of few‐time‐points renal dosimetry for [177Lu]Lu‐PSMA‐617 therapy: Analysis with nonlinear mixed‐effects modeling
Source: Med Phys. 2025 Dec 25;53(1):e70212. doi: 10.1002/mp.70212 (PMC12740544; doi:10.1002/mp.70212)
Supplement: Supplementary file 1 — Supporting Information [file MP-53-0-s001.pdf]

Supplemental File

Accuracy and Precision of Few-Time-Points Renal Dosimetry for [^{177}Lu]Lu-PSMA-617 Therapy: Analysis with Non-linear Mixed-Effects Modeling

Assyifa R. Hakim[1,2$], Deni Hardiansyah[1,2*$], Elham Yousefzadeh-Nowshahr[2,3], Ursula Nemer[4], Rien Ritawidya[1,5], Felix Kind[4], Ambros J. Beer[3], Philipp T. Meyer[4], Gerhard Glatting[2,3#], Michael Mix[4#]

[1]Medical Physics and Biophysics, Physics Department, Faculty of Mathematics and Natural Sciences, Universitas Indonesia, Depok, 16424, Indonesia

[2]Medical Radiation Physics, Department of Nuclear Medicine, Ulm University, Ulm, 89081, Germany

[3]Department of Nuclear Medicine, Ulm University, Ulm, 89081, Germany

[4]Department of Nuclear Medicine, Medical Center – University of Freiburg, Faculty of Medicine, University of Freiburg, 79106, Germany

[5]Research Center for Radioisotope, Radiopharmaceutical, and Biodosimetry Technology, National Research and Innovation Agency (BRIN), Tangerang Selatan, 15314, Indonesia

[$]equal contribution

*Corresponding Author

[#]Shared senior authorship

Nonlinear Mixed Effects Modelling

This study used the Sum-of-Exponentials Function (SOEF) with six parameters as follows

$$f(t) = A_1 e^{-(\lambda_1+\lambda_{phys})t} + A_2 e^{-(\lambda_2+\lambda_{phys})t} - A_3 e^{-(\lambda_3+\lambda_{phys})t} - (A_1 + A_2 - A_3) e^{-(\lambda_{bc}+\lambda_{phys})t} \quad (1)$$

where A_k ($k = 1, 2, and$ 3) are the coefficients for the respective exponential terms, λ_k ($k = 1, 2, and$ 3) describes the biological uptake or clearance rates of the radiopharmaceutical in the kidneys, λ_{phys} describes the physical decay constant of ^{177}Lu which is $\frac{\ln(2)}{T_{\frac{1}{2}}}$ where $T_{\frac{1}{2}}$ is the half-life of ^{177}Lu (6.6443 days)[1], and λ_{bc} describes the uptake rate due to the blood circulation with a half-life of 1 minute $\left(\lambda_{bc} = \frac{\ln(2)}{1\ minute}\right)$.[2] The estimated parameters (A_i and λ_i) are constrained to have a value greater than zero.

The NLME framework describes the data as the model function with some residual errors

$$y_i = f(t_i) + g(\varepsilon_i) \quad (2)$$

where y_i and t_i are the data and time at i-th time point respectively, and $g(\varepsilon_i)$ is the corresponding residual error function which ε_i is normally distributed with mean zero and variance Σ^2 ($\varepsilon_i \sim \mathcal{N}(0, \Sigma^2)$)[3]. In this study, a proportional residual error model was used. It is described as

$$g(\varepsilon_i) = f(t_i) \times \varepsilon_i \text{ with} \quad (3)$$

$$\varepsilon_i = \varsigma^2 \times \epsilon_i \quad (4)$$

where ς is the fractional standard deviation and the value ϵ_i is the errors which are distributed normally with mean zero and the variance of one ($\epsilon_i \sim \mathcal{N}(0, 1)$).[2,4] The parameters of the model are further represented with

$$p = TV_p \times \exp(\eta_{p,n}) \quad (5)$$

where p is a model parameter. TV_p is the parameter's typical value which is part of the fixed effect of the parameter, it has the same value for all subjects. $\eta_{p,n}$ is the random effect of the parameter p in subject n which describe the variability between subjects, this value is distributed normally with mean zero and variance Ω_p^2 ($\eta_{p,n} \sim \mathcal{N}(0, \Omega_p^2)$).[3] This NLME framework performs curve fitting to all subjects in the population and estimates the population parameters (in this case, TV_p and Ω_p^2 for all parameters and σ^2).[3,5]

Uncertainty of RMSE and MAPE

The relative deviation between (RD) eTIA and rTIA for method k and patient m can be evaluated by

$$RD_{k,m} = \frac{eTIA_{k,m} - rTIA_m}{rTIA_m} \tag{6}$$

Since both eTIA and rTIA have standard deviation (SD), we can calculate the uncertainty value of individual RD as

$$\Delta RD_{k,m} = \sqrt{\left(\frac{\partial RD_{k,m}}{\partial \mathrm{eTIA}_{k,m}} SD\mathrm{eTIA}_{k,m}\right)^2 + \left(\frac{\partial RD_{k,m}}{\partial \mathrm{rTIA}_{k,m}} SD\mathrm{rTIA}_{k,m}\right)^2} \tag{7}$$

$$\Delta RD_{k,m} = \sqrt{\left(\frac{SD\mathrm{eTIA}_{k,m}}{\mathrm{rTIA}_{k,m}}\right)^2 + \left(\frac{\mathrm{eTIA}_{k,m} \times SD\mathrm{rTIA}_{k,m}}{\left(\mathrm{rTIA}_{k,m}\right)^2}\right)^2} \tag{8}$$

The mean of RD over all patients can be calculated by

$$MeanRD_k = \frac{1}{m}\sum_{i=1}^{m} RD_{k,i} \tag{9}$$

The uncertainty of this value can also be evaluated by taking the error propagation

$$\Delta MeanRD_k = \sqrt{\sum_{i=1}^{m}\left(\frac{\partial MeanRD_k}{\partial RD_{k,i}} \Delta RD_{k,i}\right)^2} \tag{10}$$

$$\Delta MeanRD_k = \sqrt{\sum_{i=1}^{m}\left(\frac{\Delta RD_{k,i}}{m}\right)^2} \tag{11}$$

The SD value of RD can be calculated by

$$SDRD_k = \sqrt{\sum_{i=1}^{m}\left(\frac{RD_{k,i} - MeanRD_k}{m-1}\right)^2} \tag{12}$$

The uncertainty for this SD value can be evaluated by

$$\Delta SDRD_k = \sqrt{\sum_{i=1}^{m}\left(\frac{\partial SDRD_k}{\partial RD_{k,i}} \Delta RD_{k,i}\right)^2} \tag{13}$$

$$\Delta SDRD_k = \sqrt{\sum_{i=1}^{m}\left(\frac{RD_{k,i} - MeanRD_k}{(m-1) \times SDRD_k} \times \Delta RD_{k,i}\right)^2} \tag{14}$$

The root-mean-square error (RMSE) can be calculated by

$$RMSE_k = \sqrt{(SDRD_k)^2 + (MeanRD_k)^2} \tag{15}$$

Therefore, the uncertainty of RMSE can be calculated by

$$SD_{RMSE_k} = \sqrt{\left(\frac{\partial RMSE}{\partial MeanRD_k} \Delta MeanRD_k\right)^2 + \left(\frac{\partial RMSE}{\partial SDRD_k} \Delta SDRD_k\right)^2} \tag{16}$$

$$SD_{RMSE_k} = \sqrt{\left(\frac{MeanRD_k}{RMSE}\Delta MeanRD_k\right)^2 + \left(\frac{SDRD_k}{RMSE}\Delta SDRD_k\right)^2} \quad (17)$$

The Mean Absolute Percentage Error (MAPE) can be evaluated by

$$MAPE_k = \frac{1}{m}\sum_{i=1}^{m}\left|\frac{eTIA_{k,m} - rTIA_m}{rTIA_m}\right| \quad (18)$$

The uncertainty value can be calculated as

$$SD_{MAPE_k} = \sqrt{\left(\frac{\partial MAPE}{\partial eTIA}SDeTIA\right)^2 + \left(\frac{\partial MAPE}{\partial rTIA}SDrTIA\right)^2} \quad (19)$$

$$SD_{MAPE_k} = \sqrt{\sum_{i=1}^{m}\left(\frac{SD\mathrm{eTIA}_{k,i}}{m\times \mathrm{rTIA}_{k,i}}\right)^2 + \sum_{i=1}^{m}\left(\frac{\mathrm{eTIA}_{k,i}\times SD\mathrm{rTIA}_{k,i}}{m\times\left(\mathrm{rTIA}_{k,i}\right)^2}\right)^2} \quad (20)$$

Table S1. Accuracy (RMSEs, MAPEs, RD5, RD10, and RD20) and precision (distribution of individual CVs) of eTIAs from NTP and all TP combinations of FTP with NLMEM TIA calculations. Bold indicates the lowest RMSE and MAPE for each FTP combination. Standard deviations (SDs) of RMSE and MAPE were derived by error propagation from individual RD values. RD5, RD10, and RD20 represent the counts of patients with RD values exceeding 5%, 10%, and 20%, respectively, with the values in parentheses indicating the percentage of patients above each threshold. N is the number of data points, and K is the total number of estimated parameters, including the 6 SOEF typical value parameters, 6 inter-individual variability components, and 1 residual error term.

**FTP Combination**	$\frac{N}{K}$	**Combination of Time Points**	**RD [%]**		**CV [%]**		**RMSE (SD)**	**MAPE (SD)**	**RD5 (%)**	**RD10 (%)**	**RD20 (%)**
			**Mean (SD)**	**Median [Min, Max]**	**Mean (SD)**	**Median [Min, Max]**					
NTPme	-	-	22.1 (61.4)	7.2 [-55.7, 303.8]	27.5 (0.0)	27.5 [27.5, 27.5]	65.3 (9.3)	42.2 (4.8)	61 (97)	52 (83)	34 (54)
NTPmd	-	-	15.1 (57.9)	1.0 [-58.3, 280.6]	6.2 (0.0)	6.2 [6.2, 6.2]	59.8 (2.5)	39.4 (1.2)	58 (92)	55 (87)	39 (62)
STP	$\frac{311}{13}$	TP1	1.9 (23.9)	-0.2 [-43.6, 79.7]	33.1 (5.0)	32.4 [22.7, 48.2]	24.0 (6.1)	17.1 (4.6)	47 (75)	35 (56)	25 (40)
		TP2	0.0 (14.3)	0.1 [-43.0, 36.3]	21.2 (4.3)	19.7 [14.7, 30.4]	14.3 (2.9)	10.1 (2.8)	40 (63)	23 (37)	9 (14)

		**TP3**	**1.2 (10.9)**	**0.4 [-39.0, 44.2]**	17.1 (4.9)	16.1 [10.9, 42.4]	**11.0 (2.5)**	**7.0 (2.3)**	**31 (49)**	**15 (24)**	**3 (5)**
		TP4	0.8 (13.4)	-0.5 [-32.1, 38.6]	20.0 (19.6)	16.1 [10.6, 168.7	13.4 (3.2)	9.3 (3.5)	34 (54)	22 (35)	7 (11)
		TP5	4.4 (32.5)	-1.2 [-45.5,136.0]	31.8 (10.9)	31.0 [16.1, 88.0]	32.8 (9.1)	21.5 (5.0)	46 (73)	36 (57)	27 (43)
2TP	$\frac{312}{13}$	TP12	-0.5 (14.3)	0.0 [-43.3, 33.5]	20.9 (4.8)	19.5 [14.3, 35.9]	14.3 (2.7)	9.7 (2.8)	31 (49)	23 (37)	11 (17)
		TP13	1.0 (10.1)	0.7 [-42.4, 40.7]	18.3 (44.2)	12.6 [8.3, 363.4]	10.2 (1.9)	5.9 (6.0)	25 (40)	10 (16)	3 (5)
		TP14	-0.3 (8.5)	-0.3 [-32.9, 28.6]	19.0 (56.1)	10.5 [7.6, 450.6]	8.5 (2.0)	5.3 (7.3)	24 (38)	8 (13)	2 (3)
		TP15	0.8 (16.6)	-1.1 [-28.9, 55.7]	22.0 (7.4)	20.9 [9.6, 48.0]	16.6 (3.5)	11.5 (3.0)	40 (63)	26 (41)	13 (21)
		TP23	1.8 (11.0)	0.5 [-42.3, 30.8]	16.9 (9.9)	14.6 [10.9, 81.9]	11.1 (2.3)	7.3 (2.6)	31 (49)	19 (30)	2 (8)
		TP24	0.1 (8.9)	0.3 [-33.9, 16.7]	25.3 (90.9)	10.3 [7.8, 701.5]	8.9 (5.9)	6.3 (12.1)	28 (44)	15 (24)	1 (2)

		**TP25**	**-0.4 (6.3)**	**-0.3 [-14.9, 17.5]**	8.5 (2.4)	7.8 [5.7, 20.7]	**6.3 (1.6)**	**4.8 (1.3)**	**29 (46)**	**5 (8)**	**0 (0)**
		TP34	1.3 (10.9)	0.4 [-31.9, 41.3]	14.9 (5.4)	13.5 [8.6, 43.0]	11,0 (2.4)	7.2 (2.1)	29 (46)	15 (24)	5 (8)
		TP35	1.5 (8.3)	0.1 [-20.3, 28.3]	12.4 (3.0)	11.9 [6.8, 24.4]	8.4 (2.0)	5.8 (1.8)	27 (43)	13 (21)	2 (3)
		TP45	2.7 (14.1)	0.1 [-31.5, 43.1]	17.3 (4.7)	15.9 [10.4, 31.8]	14.3 (3.3)	9.4 (2.5)	33 (52)	20 (32)	9 (14)
3TP	$\frac{313}{13}$	TP123	1.0 (9.3)	0.1 [-41.7, 34.7]	35.7 (182.9)	12.0 [8.3, 1464.7]	9.4 (31.8)	5.7 (26.1)	24 (38)	9 (14)	3 (5)
		TP124	-1.1 (7.7)	-0.1 [-36.4, 16.1]	14.5 (45.2)	8.5 [5.9, 367.5]	7.7 (2.8)	4.4 (6.1)	19 (30)	10 (16)	1 (2)
		TP125	-0.7 (4.6)	0.0 [-10.4, 13.3]	20.7 (104.6)	7.3 [4.7, 837.6]	4.7 (18.7)	3.2 (12.3)	17 (27)	4 (6)	0 (0)
		TP134	0.2 (8.0)	0.0 [-36.9, 25.0]	13.1 (35.3)	8.3 [6.0, 289.1]	8.0 (1.4)	4.7 (4.8)	20 (32)	9 (14)	2 (3)
		**TP135**	**0.8 (3.9)**	**0.1 [-7.6, 20.7]**	6.3 (0.9)	6.2 [4.9, 9.8]	**3.9 (1.1)**	**2.3 (1.0)**	**8 (13)**	**1 (2)**	**1 (2)**

		TP145	0.5 (7.0)	-0.2 [-11.6, 26.0]	9.4 (2.7)	8.7 [5.6, 17.9]	7.0 (1.5)	4.3 (1.3)	22 (35)	8 (13)	1 (2)
		TP234	-0.6 (9.1)	0.2 [-34.5, 16.6]	11.1 (2.9)	10.7 [7.9, 28.3]	9.1 (1.5)	6.4 (1.5)	31 (49)	16 (25)	1 (2)
		TP235	0.3 (4.8)	0.1 [-14.0, 10.3]	11.8 (37.1)	6.9 [5.4, 301.2]	4.8 (4.0)	3.7 (4.7)	20 (32)	2 (3)	0 (0)
		TP245	-0.3 (4.5)	0.1 [-13.5, 10.5]	14.3 (26.6)	7.2 [5.1, 162.9]	4.5 (4.2)	3.4 (3.7)	16 (25)	2 (3)	0 (0)
		TP345	2.5 (9.2)	0.8 [-22.0, 26.5]	26.5 (80.7)	11.6 [7.8, 513.0]	9.5 (8.2)	6.6 (10.4)	28 (44)	16 (25)	3 (5)
4TP	$\frac{314}{13}$	TP1234	-0.6 (7.5)	-0.1 [-35.8, 15.6]	10.4 (14.3)	8.2 [5.7, 121.2]	7.5 (1.5)	4.5 (2.3)	19 (30)	9 (14)	1 (2)
		**TP1235**	**0.0 (1.4)**	**0.0 [-5.7, 2.7]**	5.1 (0.6)	5.1 [4.0, 6.4]	**1.4 (0.8)**	**0.9 (0.8)**	**1 (2)**	**0 (0)**	**0 (0)**
		TP1245	-0.5 (1.4)	-0.1 [-4.7, 2.9]	5.2 (0.9)	5.2 [4.1, 10.5]	1.5 (0.8)	0.9 (0.9)	0 (0)	0 (0)	0 (0)
		TP1345	0.9 (4.0)	0.2 [-6.3, 21.1]	5.8 (1.2)	5.5 [4.1, 12.6]	4.1 (1.0)	2.4 (0.9)	10 (16)	1 (2)	1 (2)

		TP2345	0.0<br>(4.3)	0.1<br>[-13.5, 10.6]	18.7<br>(67.1)	6.7 [5.0,<br>443.6]	4.3<br>(9.4)	3.2<br>(8.5)	14<br>(22)	2<br>(3)	0<br>(0)

References

1. Kondev FG. Nuclear Data Sheets for A=177. *Nuclear Data Sheets*. 2019;159:1-412. doi:10.1016/j.nds.2019.100514

2. Hardiansyah D, Riana A, Beer AJ, Glatting G. Single-time-point dosimetry using model selection and nonlinear mixed-effects modelling: a proof of concept. *EJNMMI Phys*. 2023;10(1). doi:10.1186/s40658-023-00530-1

3. Bonate PL. *Pharmacokinetic-Pharmacodynamic Modeling and Simulation*. Springer US; 2011. doi:10.1007/978-1-4419-9485-1

4. Hardiansyah D, Yousefzadeh-Nowshahr E, Kind F, et al. Single-Time-Point Renal Dosimetry Using Nonlinear Mixed-Effects Modeling and Population-Based Model Selection in [177 Lu]Lu-PSMA-617 Therapy. *J Nucl. Med*. 2024;65(4):566-572. doi:10.2967/jnumed.123.266268

5. Bach T, An G. Comparing the performance of first-order conditional estimation (FOCE) and different expectation–maximization (EM) methods in NONMEM: real data experience with complex nonlinear parent-metabolite pharmacokinetic model. *J Pharmacokinet Pharmacodyn*. 2021;48(4):581-595. doi:10.1007/s10928-021-09753-0
